# A Hybrid Model Based on Improved Transformer and Graph Convolutional Network for COVID-19 Forecasting

**DOI:** 10.3390/ijerph191912528

**Published:** 2022-09-30

**Authors:** Yulan Li, Kun Ma

**Affiliations:** 1 Faculty of Civil Engineering and Mechanics, Kunming University of Science and Technology, Kunming 650500, China; 2 Faculty of Science, Kunming University of Science and Technology, Kunming 650500, China

**Keywords:** COVID-19, hybrid model, prediction, deep learning

## Abstract

The coronavirus disease 2019 (COVID-19) has spread rapidly around the world since 2020, with a significant fatality rate. Until recently, numerous countries were unable to adequately control the pandemic. As a result, COVID-19 trend prediction has become a hot topic in academic circles. Both traditional models and existing deep learning (DL) models have the problem of low prediction accuracy. In this paper, we propose a hybrid model based on an improved Transformer and graph convolution network (GCN) for COVID-19 forecasting. The salient feature of the model in this paper is that rich temporal sequence information is extracted by the multi-head attention mechanism, and then the correlation of temporal sequence information is further aggregated by GCN. In addition, to solve the problem of the high time complexity of the existing Transformer, we use the cosine function to replace the softmax calculation, so that the calculation of query, key and value can be split, and the time complexity is reduced from the original $O(N^{2})$ to O(N). We only concentrated on three states in the United States, one of which was the most affected, one of which was the least affected, and one intermediate state, in order to make our predictions more meaningful. We use mean absolute percentage error and mean absolute error as evaluation indexes. The experimental results show that the proposed time series model has a better predictive performance than the current DL models and traditional models. Additionally, our model’s convergence outperforms that of the current DL models, offering a more precise benchmark for the control of epidemics.

## 1. Introduction

Novel coronavirus pneumonia, referred to as COVID-19, is an acute infectious pneumonia caused by a novel coronavirus, which is highly infectious, and to which the population is generally susceptible. It is of great significance to study the development trend of COVID-19 and build a reasonable prediction model for the scientific and effective prevention and control of the COVID-19 epidemic [1]. Specifically, COVID-19 follows specific patterns that are based on the dynamic spread of the epidemic. When it occurred, alternative measures using different methods were used to detect and assess this type of epidemic. Any epidemic that occurs in a state or country has varying temporal magnitudes—especially in terms of changing weather cycles and virus transmission over time—and is non-linear in nature. Researchers have devised non-linear systems to describe the suddenness of infectious diseases in order to capture these non-linear and striking variations [2,3].

At present, experts and scholars at home and abroad have established relevant prediction models for COVID-19. For example, Autoregressive Integrated Moving Average Model (ARIMA), Seasonal Autoregressive Integrated Moving Average (SARIMA), epidemic dynamics model, Long and Short-term Memory (LSTM) model, Gate Recurrent Unit (GRU) model, etc. Among them, ARIMA is the most representative [4,5,6]. It has a high degree of fitting for linear data, but it cannot predict nonlinear data well. Under normal circumstances, time series data contain linear and nonlinear parts, so a linear prediction of data solely through the ARIMA model often cannot meet people’s needs [7]. The LSTM model is widely used in stock prediction, second-hand housing transaction prediction, and other fields [8]. It has a good effect on solving nonlinear time series problems but also has overfitting, gradient disappearance, hidden layer selection, and other problems. It requires a large amount of data and has high complexity and uncertainty. The traditional single model is mainly for causality and time series model analysis, which cannot extract more comprehensive data information. At present, the mainstream prediction methods can be divided into several prediction methods based on the dynamic differential equation model, regression model, intelligent algorithms, and Deep Learning (DL) models, showing a general trend of development from the simple regression model to complex DL models [9]. Most dynamical differential equation models do not take human factors into consideration and describe the process prediction results of the natural transmission of disease, which show disparity with reality [10,11]. The time series model is suitable for the prediction disease transmission that cannot be determined by the way and mode of infection. It needs to provide detailed incidence data, which is feasible and frequently used at present. Multiple regression is often used to analyze the impact of multiple factors and analyze the complex characteristics of the epidemic of infectious diseases with high prediction accuracy [12]. However, because data such as regions and diseases must be adjusted to the current situation in practice, this method’s popularity is limited. As a result, using effective prediction models to accurately predict the number of COVID-19 infections is crucial for government policymakers.

Researchers domestically and internationally have developed COVID-19 trend prediction models based on DL since its introduction. These algorithms have helped scientific research organizations and medical experts anticipate COVID-19 with accuracy. Building models to examine various scenarios and forecast the epidemic’s development pattern is a common practice among academics [13,14]. By capturing the point-wise relationship through the attention mechanism, transformer time series prediction can produce positive results, but there are still some significant flaws. Since the distribution of the sequence could change over time, the model needs to be more extrapolation-capable. Specifically, the Transformer architecture has achieved good results in the field of DL. The encoder encodes the input data, which are then decoded by the decoder network to produce the desired output based on the encoded input. As these networks have a better understanding of context, they provide a better performance. The attention and softmax mechanisms of the Transformer dot product are key to capturing long-distance tasks. Unfortunately, the spatial and temporal complexity of the Transformer dot product is limited by sequence length, especially for long-distance tasks. The convolution operation in Graph Convolutional Network (GCN) is concerned with the hidden state update of each node and has a good performance in the calculation of graph structure. However, the overall performance of the existing model in the prediction problem still has significant room for improvement, and the manner of encoding and decoding still needs to be improved [15].

In this paper, motivated by the recent progress of Transformer and GCN architecture for COVID-19 forecasting, we designed a novel network model to further improve the prediction accuracy of the model in the prediction of COVID-19. The contributions of this work are three-fold: Firstly, we propose a hybrid model based on an improved Transformer and GCN for COVID-19 forecasting. The salient feature of the model in this paper is that rich temporal sequence information is extracted by the multi head attention (MHA) mechanism, and then the correlation of temporal sequence information is further aggregated by GCN. Secondly, in order to solve the problem of the high time complexity of the existing Transformer, we use the cosine function to replace the softmax calculation, so that the calculation of K, Q, and V can be split, and the time complexity is reduced from the original $O(N^{2})$ to O(N). Finally, we empirically demonstrate that the prediction accuracy and model stability of the proposed model surpass the existing DL prediction models and traditional models. Our model prediction results can be useful for infectious disease control and related policy development.

This paper is structured as follows. Section 2 examines relevant work. The COVID-19 training data and the measurement of prediction accuracy are all covered in Section 3. The results of the experiment are presented in Section 5. This paper is concluded in Section 6.

## 2. Literature Reviews

In order to minimize the negative impact of the epidemic; contain the spread of the virus source in a timely manner before the development of the epidemic; evacuate and control the surrounding people who may be exposed to the virus source, also in a timely manner; and prevent the second outbreak of the epidemic, many scholars use relevant mathematical theories to build models to analyze different situations and predict the development trend of the epidemic [16,17,18,19]. In a time series forecast, Roy et al. [20] analyzed cumulative confirmed cases of COVID-19 in states with a high daily incidence in India. Their study may be useful as a reference to understanding risk attitudes and social media interactions across countries for more in-depth studies. For a more accurate prediction of prevalence, active cases, recovery, and death figures connected to the COVID-19 outbreak in Pakistan, Alabdulrazzaq et al. [21] suggested using a more practical Kalman filter technique in the ARIMA model. Katoch and Sidhu [22] used the ARIMA model to conduct a study that has significant promise for planning and decision making, in order to restrict the spread of the epidemic in India and provide objective projections of confirmed cases in the next days based on COVID-19 incidence in the relevant districts.

In the field of DL, there are numerous methods for predicting sexually transmitted diseases. These methods can be divided into qualitative and quantitative prediction based on their respective hypotheses. Naturally, some techniques combine the two processes for a more complete prediction. The most significant machine learning prediction models for COVID-19 were reviewed and briefly analyzed by Rahimi et al. [23].

Li et al. [24] integrated Transformer and GCN for COVID-19 forecasting, providing a new prediction model for COVID-19 prevention. However, they do not consider the time complexity of the Transformer, and the model is not compared with traditional methods. To predict COVID-19 viral evolution in the population, Miralles-pechuan et al. [25] created the SEIR epidemiological model and combined deep Q-learning and a genetic algorithm. The survey conducted by Shorten et al. [26] examined how DL could be used to combat the COVID-19 pandemic and makes suggestions for future studies. A new time series prediction method that can produce more precise predictions over a wider time range than earlier approaches was proposed by Farsani et al. [27]. The performance of the self-attention-based Transformer neural network model is comparable to other tools in terms of predicting time series issues. A new machine learning-based framework developed by La et al. [28] is capable of predicting the parameters of any epidemiological model, such as exposure and recovery rates, based on static and dynamic site features. Using GCN and LSTM in conjunction with mobile data from a graph sequence, the model infers the parameters of the SIR and SIRD models.

## 3. Datasets

To make our predictions more useful, we only focused on three states in the United States (US), covering one of the most affected states, one of the least affected states, and one intermediate state. The three states are New York (NY), Virginia (VA), and California (CA). We mainly concentrated on forecasting the quantity of confirmed cases, deaths, and vaccines. For our research, we consider two datasets. Cases and deaths are the first, and vaccinations are the second. All data sets are publicly selected, and model testing in three states with different influence can show the performance of the model prediction in this paper.

### 3.1. Confirmed Cased and Deaths Datasets

A dataset from *The New York Times* was used [29]. This 16-month data series, which ran from January 2020 to 5 May 2021, was updated every day. As of 22 April 2021, there were about 418 data points in the index. The following US states are represented by information in this dataset:

- Date: Observation date in mm/dd/yyyy.
- State: State of the USA.
- Cases: Cumulative counts of coronavirus cases till that date.
- Deaths: Cumulative counts of coronavirus deaths till that date.

A sample of the dataset is shown in Table 1. A similar dataset [30] exists, but it is not continuously updated and was only updated until February 2021. Due to this, we selected this option. The New York Times keeps this dataset after converting it to sliding window blocks. We split the dataset into training and testing portions in an 80:20 ratio. The initial 80% of the data are used for training, and the final 20% are used for evaluation.

### 3.2. Vaccinations Dataset

We vaccinated states using a global dataset provided in [31]. These time series data span the period from 13 January 2021 to 5 May 2021. They have the following features and are updated each day:

- Date.
- State name.
- Daily count of vaccinations.

Given that it only includes data for three months, this dataset is quite small. In order to increase the number of samples and ensure we had enough time to evaluate the dataset, we decided to split the dataset into training and test datasets that were split 80:20.

## 4. Methods

### 4.1. Data Preprocessing

Data for COVID-19 were initially sourced from an open-source dataset. In order to obtain regular time series data, we secondly performed data pre-processing operations on the infection rate data, such as outlier processing and null processing. One data sample’s first month’s worth of data were used as the sample, and the remaining data served as the label data. Finally, these data samples are fed into a training model that can forecast data based on the first 80% of the data for the purpose of forecasting future data based on the first 80% of the data.

### 4.2. Model

**Figure 1:**
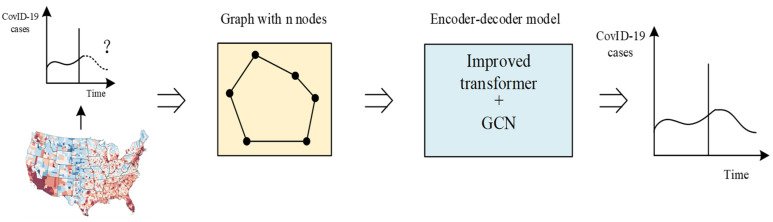
Framework of model.

In this paper, we propose a model based on the Transformer [32] and GCN [33]. The salient feature of the model in this paper is that rich temporal sequence information is extracted by the multi-head attention mechanism, and then the correlation of temporal sequence information is further aggregated by GCN. In addition, in order to solve the problem of the high time complexity of the existing Transformer, we use the cosine function to replace the softmax calculation, so that the calculation of K, Q, and V can be split, and the time complexity is reduced from the original $O(N^{2})$ to O(N). Our model not only ensures the prediction accuracy, but also obtains better prediction results with low time complexity. At the same time, the addition of GCN can better extract the spatial features of the time series, which overcomes the problem of the incomplete representation of information from a single model. The model framework is shown in Figure 1, and the following information introduces related concepts and basic knowledge. In short, our model converts historical data into graph data, and then encodes time series information through the improved Transformer model and decoded time series information by GCN, finally obtaining the prediction results of pneumonia-related problems in COVID-19 in an end-to-end manner.

#### 4.2.1. Encoder

It is common knowledge that Positional Encoding (PE) can add location information to the model and enhance its representation ability. Similar to [34], this paper adopts Dynamic Positional Encoding (*DPE*) to enhance the representational ability of the model. *DPE* can encode the embedding of the initial starting information in a cyclic and dynamic manner, overcoming the classic position-coding operation’s limitation of only being applicable to linear sequences and effectively retrieving the position information of dynamic nodes. Here, *DPE* is defined as:

(1) $$DPE_{t,i}=\left\{\begin{array}{l} \sin\left(2\pi f_{i}t+\frac{2\pi }{\omega_{d}i}\right),\begin{array}{ccc} i & is & odd \end{array}\\ \cos\left(2\pi f_{i}t+\frac{2\pi }{\omega_{d}i}\right),\begin{array}{ccc} i & is & even \end{array} \end{array}\right.$$

where

(2) $$f_{i}=\frac{{10,000}^{\frac{d}{2i}}}{2\pi }$$

(3) $$\omega_{d}=\left\{\begin{array}{cc} \frac{3\left[d/3\right]+1}{d}\left(N-N^{\frac{1}{\left[d/2\right]}}\right)+\frac{1}{\left[d/2\right]}, & \begin{array}{cc} if & d<\left[\frac{d}{2}\right] \end{array}\\ N & ,otherwise \end{array}\right.$$

$DPE_{t,i}\in R^{d}$, t is the location of the node, and d=128 is the embedding dimension, $i\in \left\{1,2,,\ldots ,n\right\}$; the angular frequency $\omega_{d}$ decreases along the dimension to lengthen the wavelength within the range N.

**Figure 2:**
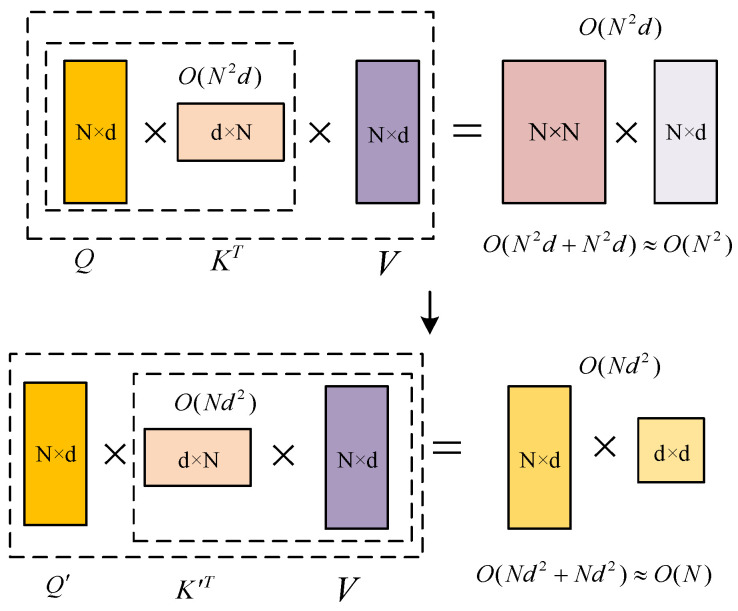
A brief analysis of time complexity.

The existing Transformer model has a high time complexity, and the realization and solution of the model require many computing resources. MHA is the key to capturing global information, but its time and space complexity is limited by sequence length, especially in the large-scale node training. Among them, softmax is the main difficulty in optimizing complexity. The calculation of *Q*, *K*, and *V* is not separable, so *Q* and *K* can only be calculated first, and then the obtained matrix can be calculated with V. The time complexity is square with the input sequence N, that is, the time complexity is $O(N^{2})$. Therefore, this paper considers a linear method (linear with the input sequence length N) to replace the softmax calculation without losing the Transformer performance. In this way, the combination rate can be used to calculate *K* and *V* first, and then with *Q*, while retaining the key characteristics of MHA. The time complexity is reduced to O(N). When the input sequence length meets N≫d, the relationship between time complexity and input sequence length is obvious, as shown in Figure 2.

The calculation of *Q*, *K*, and *V* in the previous work can be expressed as:

(4) $$H^{l}=soft\max(\frac{Q^{l}K^{l^{T}}}{\sqrt{d}})V^{l}\in R^{(n+1)\times d}$$

In this paper, *Q* and *K* are first mapped to $Q^{\prime }$ and $K^{\prime }$ by the Rectified Linear Unit (ReLU) function, which guarantees the non-negativity of $Q^{\prime }$ and $K^{\prime }$. Then, $Q^{\prime }$,$K^{\prime }$, and V are calculated by the cosine function, which transforms the softmax indivisible calculation form into three matrix dot products and swaps the order of matrix calculation according to the combination law, thus reducing the time complexity. The method for calculating the weights of the cosine function is defined as:

(5) $$f(Q_{i}^{\prime },K_{j}^{\prime })=Q_{i}^{\prime }K_{j}^{\prime }{}^{T}\cos(\frac{\pi }{2}\times \frac{i-j}{M})=Q_{i}^{\prime }K_{j}^{\prime }{}^{T}(\cos(\frac{\pi i}{2M})\cos(\frac{\pi j}{2M})+\sin(\frac{\pi i}{2M})\sin(\frac{\pi j}{2M}))\;=(Q_{i}^{\prime }\cos(\frac{\pi i}{2M})){\left(K_{j}^{\prime }\cos(\frac{\pi j}{2M})\right)}^{T}+(Q_{i}^{\prime }\sin(\frac{\pi i}{2M})){\left(K_{j}^{\prime }\sin(\frac{\pi j}{2M})\right)}^{T}$$

where i,j=1,…,n indicates the node position, M≥n, Q′=ReLU(Q), K′=ReLU(K). We define $Q_{i}^{\cos}=Q_{i}^{\prime }\cos(\frac{\pi i}{2M})$, $Q_{i}^{\sin}=Q_{i}^{\prime }\sin(\frac{\pi i}{2M})$, $K_{j}^{\cos}=K_{j}^{\prime }\cos(\frac{\pi i}{2M})$, $K_{j}^{\sin}=K_{j}^{\prime }\sin(\frac{\pi i}{2M})$. The attention module of the MHA layer can then be calculated as:

(6) $$H^{l}=\sum_{j=1}^{N}f(Q_{i}^{\prime },K_{j}^{\prime })V_{j}=\sum_{j=1}^{N}Q_{i}^{\cos}({\left(K_{j}^{\cos}\right)}^{T}V_{j})+\sum_{j=1}^{N}Q_{i}^{\sin}({\left(K_{j}^{\sin}\right)}^{T}V_{j})$$

The improved Transformer encoding section can be expressed as:

(7) $$H^{en}=H^{l=L^{en}}\in R^{(n+1)\times d}$$

(8) $$H^{l}=\sum_{j=1}^{N}f(Q_{i}^{\prime },K_{j}^{\prime })V_{j}\in R^{(n+1)\times d}$$

(9) $$Q^{l}=H^{l}W_{Q}^{L}\in R^{(n+1)\times d},W_{Q}^{l}\in R^{d\times d}$$

(10) $$K^{l}=H^{l}W_{K}^{L}\in R^{(n+1)\times d},W_{K}^{l}\in R^{d\times d}$$

(11) $$V^{l}=H^{l}W_{V}^{L}\in R^{(n+1)\times d},W_{V}^{l}\in R^{d\times d}$$

where $W_{Q}^{l}$, $W_{K}^{l}$, and $W_{V}^{l}$ are the training parameters; i,j=1,…,n denotes the node position;$H^{en}$ is a matrix containing the encoded nodes; and $Q^{l}$, $K^{l}$, and $V^{l}$ are the query, key, and value vectors of the self-attentive mechanism, respectively.

#### 4.2.2. Decoder

The Transformer architecture only uses a self-attentive mechanism for decoding and does not process the encoded feature vectors, so we consider aggregating the feature vectors in the high-dimensional space directly by GCN in the decoding stage to further enhance the feature information of the nodes in the high-dimensional space. The reason for the direct application is that the Transformer maps the nodes into a 512-dimensional vector, which is equivalent to the initial features of the GCN. With this approach, the robustness of the model is substantially increased, while the prediction accuracy can be significantly improved.

For graphs with extremely large node degree distributions, Kipf and Welling [35] limit the layer-wise convolution process to *K* = 1 to address the issue of overfitting on local neighborhood structures. It further approximates λ≈2 and the equation simplifies to:

(12) $$g_{\theta^{\prime }}\cdot x\approx \theta_{0}^{\prime }x+\theta_{1}^{\prime }(L-I_{N})x=\theta_{0}^{\prime }x-\theta_{1}^{\prime }D^{-\frac{1}{2}}AD^{-\frac{1}{2}}x$$

with two free parameters, $\theta_{0}^{\prime }$ and $\theta_{1}^{\prime }$. After constraining the number of parameters with $\theta =\theta_{0}^{\prime }=-\theta_{1}^{\prime }$, we can obtain the following expression:

(13) $$g_{\theta }\cdot x\approx \theta (I_{N}+D^{-\frac{1}{2}}AD^{-\frac{1}{2}})x$$

Note that stacking this operator could lead to numerical instabilities and exploding/vanishing gradients. Kipf and Welling [35] introduce the renormalization trick: $I_{N}+D^{-\frac{1}{2}}AD^{-\frac{1}{2}}\to {\tilde{D}}^{-\frac{1}{2}}\tilde{A}{\tilde{D}}^{-\frac{1}{2}}$, with $\tilde{A}=A+I_{N}$ and ${\tilde{D}}_{ij}=\sum_{j}{\tilde{A}}_{ij}$. Finally, they [35] generalize the definition to a signal $X\in R^{N\times C}$ with C input channels and F filters for feature maps as follows:

(14) $$Z={\tilde{D}}^{-\frac{1}{2}}\tilde{A}{\tilde{D}}^{-\frac{1}{2}}X\Theta$$

where $\Theta \in R^{C\times F}$ is a matrix of filter parameters and $Z\in R^{N\times F}$ is the convolved signal matrix.

In this work, we leverage the GCN architecture introduced in [33] by defining the node features $x_{i}^{l+1}$ and edge features $e_{ij}^{l+1}$ as follows:

(15) xil+1=xil+ReLU(BN(W1lxil+∑j~iηijl⊙W2lxjl)) with ηijl=σ(eijl)∑j′~iσ(eij′l)+ε

(16) eijl+1=eijl+ReLU(BN(W3leijl+W4lxil+W5lxil))

where $W\in R^{h\times h}$, σ is the sigmoid function, ε is a small value, *ReLU* is the rectified linear unit, and *BN* stands for batch normalization. At the input layer, we have $x_{i}^{l=0}=\alpha_{i}$ and $e_{ij}^{l=0}=\beta_{ij}$.

### 4.3. Training Data

The original COVID-19 data are divided into three sets in this study: a training set and a test set, with a ratio of 8:2. Typically, the validation set is used to determine the network structure of the model and modify the hyperparameters. The validation set is primarily used to evaluate the model’s generalizability, while the training set is primarily used to train the model and choose the weight model.

### 4.4. Prediction Accuracy Measurement

Three commonly used indicators in regression prediction tasks are Mean Square Error (*MSE*), root mean square error, mean absolute error, and mean absolute percentage error (*MAPE*). *MSE* is the square of the discrepancy between an estimated and true value for a parameter. The amount of data change can be determined by *MSE*. It is common practice to use the derivative by square as the loss function in linear regression because it is straightforward to calculate. How well a prediction model can explain experimental data is gauged by the *MSE* value. The better the data, the lower the MSE value. In this paper, the model evaluation indices *MSE* and *MAPE* are used. The following formulas are used to calculate each indicator:

(17) $$MSE=\frac{1}{n}\sum_{i=1}^{n}{\left({\hat{y}}_{i}-y_{i}\right)}^{2}$$

(18) $$MAPE=\sum_{i=1}^{n}\left|\frac{{\hat{y}}_{i}-y_{i}}{{\hat{y}}_{i}}\right|\frac{100\%}{n}$$

## 5. Experimental Results

### 5.1. Model Comparison

We compare three DL models and two traditional models. We shall demonstrate that our model outperforms the competition. While our model benefits from huge datasets, the LSTM may be more useful in some situations where there is a limited amount of data. By employing *MAPE* and *MSE* score metrics to analyze the last 20% or 30% of days, we also describe how each model fits the observations differently in diagrams. In areas with high populations, such as NY, VA, and CA, training converges and trains quickly due to an existing temporal correlation throughout the days. Training errors may exist in different datasets, and the differences between models on limited datasets are not large.

**Figure 3:**
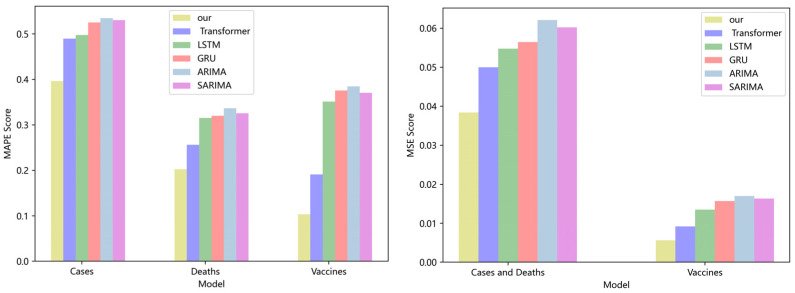
Comparison of *MAPE* and *MSE* score for CA cases, deaths, and vaccines.

Figure 3 compares four DL models and two traditional models for CA cases, deaths, and vaccinations. Our model has lower *MAPE* and *MSE* ratings than the Transformer, LSTM, and GRU. If the *MAPE* score is low, the model performs better. On the CA dataset, the *MAPE* score of our model is 19%, 23%, and 26% higher than the Transformer, LSTM, and GRU, respectively. The *MSE* score of our model is 15.6%, 20.4%, and 23.7% higher than the Transformer, LSTM, and GRU, respectively. Compared with the classical ARIMA and SARIMA models [36,37], our model also has lower *MAPE* and *MSE* values. On the CA dataset, the *MAPE* score of our model is 34% and 36% lower than that of the ARIMA and SARIMA models, respectively. The MSE score of our model is 28.3% and 33.3% lower than that of the ARIMA and SARIMA models, respectively. No significant difference can be seen in the performance of LSTM, GRU, ARIMA, and SARIMA models, because the values in the dataset are too stable, which is not conducive to the embodiment of the advantages of the model. As the calculation of model softmax can be broken down into linear complexity, and the aggregation operation of GCN further strengthens the coupling between data, the experimental results are superior to those of the prior model.

**Figure 4:**
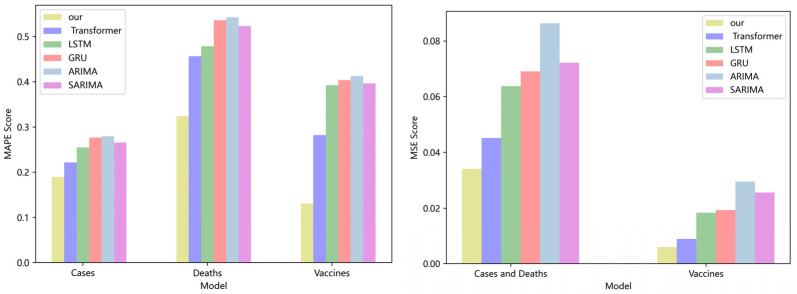
Comparison of MAPE and MSE score for NY cases, deaths, and vaccines.

A comparison of four DL models and two traditional models for NY cases, deaths, and vaccinations is shown in Figure 4. Transformers, LSTM, and GRU all have greater *MAPE* and *MSE* ratings than our model. The model is superior if the *MAPE* score is low. On the NY dataset, the *MAPE* score of our model is 8.6%, 12%, and 15.3% lower than the Transformer, LSTM, and GRU, respectively. The *MSE* score of our model is 10.8%, 12.4%, and 13.8% lower than the Transformer, LSTM, and GRU, respectively. Our model also offers lower *MAPE* and *MSE* values with more advantages when compared to the traditional ARIMA and SARIMA models. Our model’s *MAPE* score is 11% and 17% lower on the VA dataset than the scores of the ARIMA and SARIMA models, respectively. In comparison to the ARIMA and SARIMA models, our model’s *MSE* score is 15% and 21% higher, respectively. In terms of overall comparison, our model is superior.

**Figure 5:**
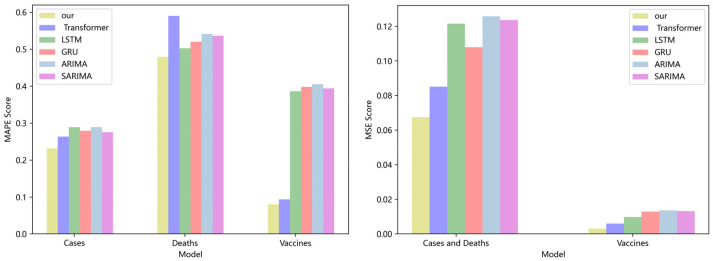
Comparison of MAPE and MSE score for VA cases, deaths, and vaccines.

Four DL models and two traditional models for NY cases, deaths, and vaccinations are compared in Figure 5. The Transformer, LSTM, and GRU all have better *MAPE* and *MSE* values than those of our model. The model is superior if the MAPE score is low. On the NY dataset, the *MAPE* score of our model is 8.6%, 12%, and 15.3% lower than the Transformer, LSTM, and GRU, respectively. The MSE score of our model is 10.8%, 12.4%, and 13.8% higher than the Transformer, LSTM, and GRU, respectively. Our approach has more advantages over the traditional ARIMA and SARIMA models and lower *MAPE* and *MSE* values. Our model’s *MAPE* score is 11% and 17% lower on the VA dataset than the scores of the ARIMA and SARIMA models, respectively. In comparison to the ARIMA and SARIMA models, our model’s *MSE* score is 15% and 21% higher, respectively. In terms of overall comparison, our model is superior. On many datasets, our model has demonstrated good prediction accuracy, which suggests that it has good characterization capabilities and efficiently captures long-range time series features using linear computational techniques.

**Figure 6:**
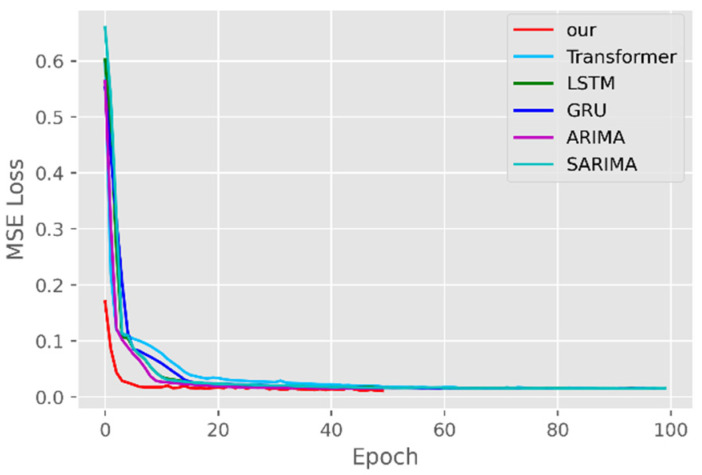
Comparison of training loss for CA cases and deaths.

**Figure 7:**
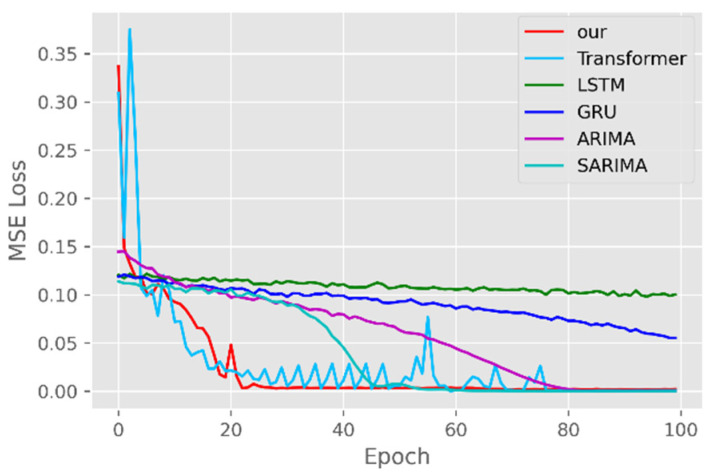
Comparison of training loss for CA vaccines.

**Figure 8:**
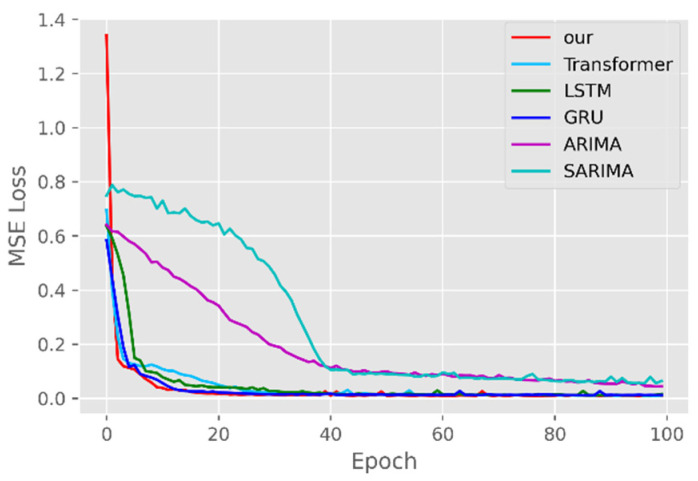
Comparison of training loss for NY cases and deaths.

**Figure 9:**
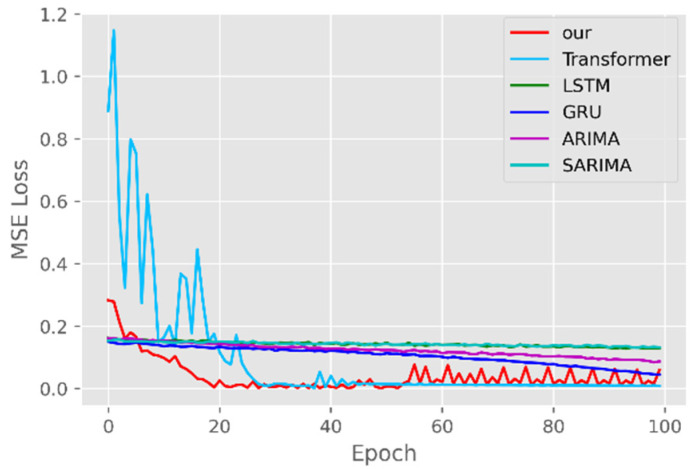
Comparison of training loss for NY vaccines.

**Figure 10:**
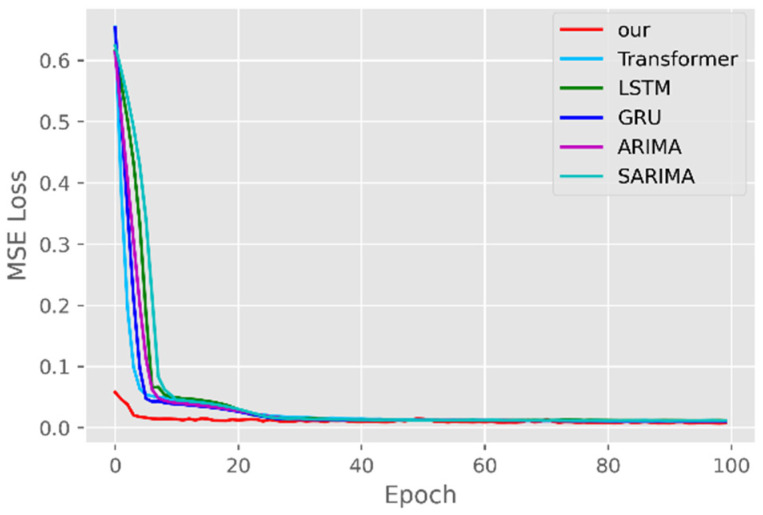
Comparison of training loss for VA cases and deaths.

**Figure 11:**
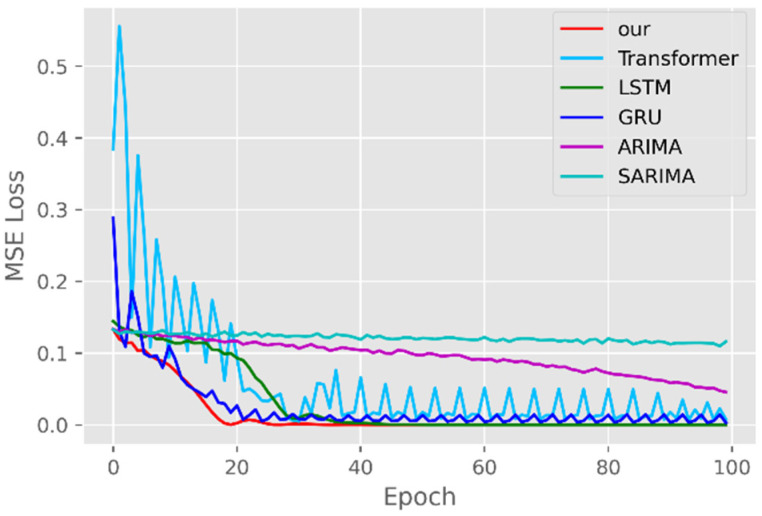
Comparison of training loss for VA vaccines.

The model training losses are compared in order to further assess the model’s propensity to predict outcomes for various sets of data. Figure 6, Figure 7, Figure 8, Figure 9, Figure 10 and Figure 11 demonstrate how well our model converges during training and outperforms the Transformer, LSTM, and GRU models in terms of overall performance. Similarly, our model has better stability than the traditional model of ARIMA and SARIMA. In particular, the ARIMA and SARIMA models showed better stability than our proposed models from epoch 55 in the NY vaccines dataset. The above phenomenon is also normal, because the data distribution between different data sets will have a great impact on the model convergence. At the same time, it also implies that the robustness of the DL model can be further enhanced. The biggest advantage of our model is that it can converge to stable values quickly. Without losing the Transformer’s performance, the linear computational approach proposed in this paper can match the state-of-the-art DL model in a very short period of time. At the same time, the hybrid coding and decoding architecture of the Transformer and GCN further enhances the robustness of the model, allowing it to maintain good stability throughout the training period, which in turn ensures the prediction results. In summary, the validity of the model in this paper is indirectly illustrated in terms of its convergence. Our model may appear to be less effective under different datasets, but this does not affect the fact that it performs better in most datasets. The occurrence of the above situation also implies that we have more room for improvement in our DL learning model, which is a positive sign.

Traditional time series forecasting methods such as the ARIMA model and SARIMA model have theoretical guarantees, but they are mainly suitable for univariate forecasting problems and require time series to be stationary, which greatly limits their application in real-world complex time series data. If there are too many problems or variables, it is difficult for the traditional timing model to have a good prediction effect and performance [38]. DL models can learn complex data representations, thereby alleviating the need for hand motion feature engineering and model design. The availability of the open-source backpropagation framework and DL framework also simplifies network training, allowing the customization of network components and loss functions. For a long time series, the forward calculation outputs all the prediction results instead of the stepwise method, which greatly improves the reasoning speed of long time series prediction. Therefore, the DL method has a better development prospect than the traditional time series model.

### 5.2. Forecasting the Number of Confirmed Cases

**Figure 12:**
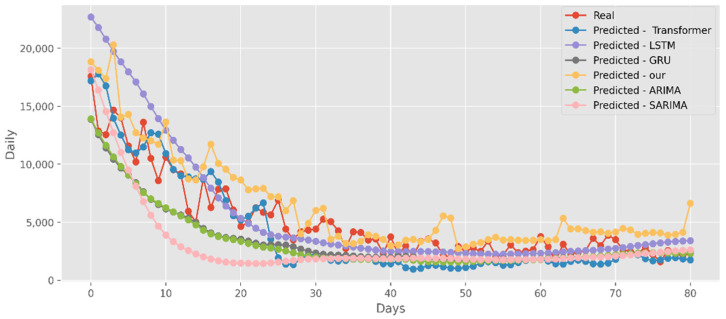
Comparison of COVID-19 prediction models of cases in CA.

**Figure 13:**
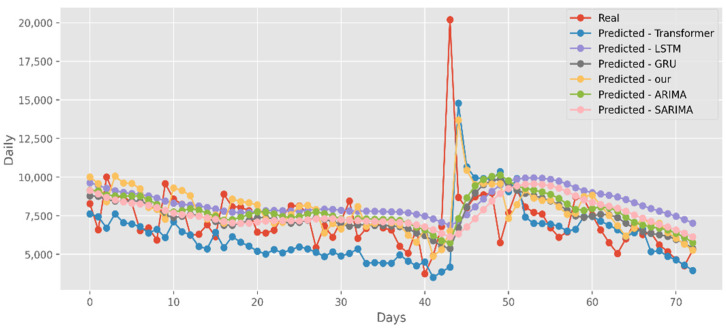
Comparison of COVID-19 prediction models of cases in NY.

**Figure 14:**
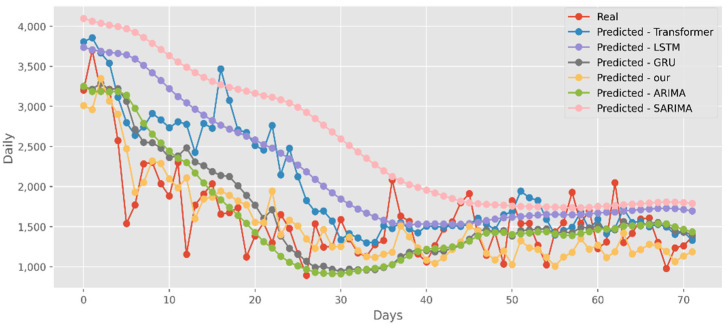
Comparison of COVID-19 prediction models of cases in VA.

The predictions made using four DL algorithms and two traditional models in relation to the actual test data set are displayed in Figure 12. The cumulative cases in CA are displayed on the graph’s *y*-axis. The latest two months, March and April 2021, are displayed on the *x*-axis. By predicting date ranges using NY test data sets, Figure 13 also compares four DL algorithms and two traditional models. NY’s daily cases are displayed on the *y*-axis. The date ranges in the VA test datasets are anticipated in Figure 14, which also compares four DL algorithms and two traditional models. Daily VA cases are displayed on the *y*-axis. The best option in this case is our model because it attempted to capture the peaks, which are essential for COVID-19 predictions. Additionally, it can be shown that the prediction curve for the model used in this study fits the true value well and fluctuates less than usual. Theoretically, the multi-head attention mechanism plus the line computation method extracts the feature-rich temporal vectors from the new crown data, and the GCN uses its own aggregation capability to further enhance the temporal information in the high-dimensional space, improving prediction accuracy and stability.

### 5.3. Forecasting the Number of Deaths

**Figure 15:**
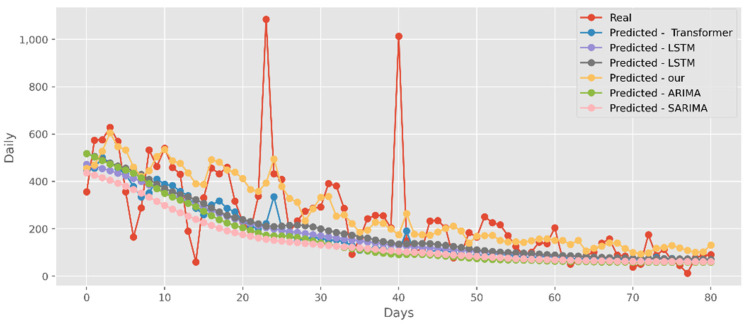
Comparison of COVID-19 prediction models of deaths in CA.

**Figure 16:**
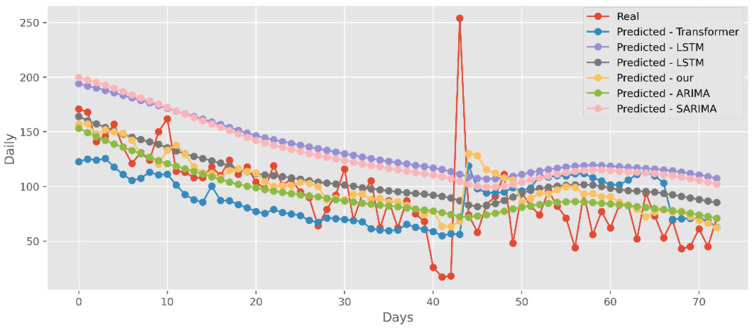
Comparison of COVID-19 prediction models of deaths in NY.

**Figure 17:**
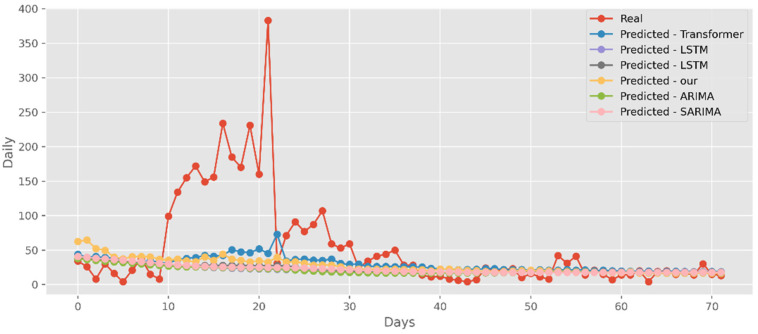
Comparison of COVID-19 prediction models of deaths in VA.

Similar to the analysis above, our model fits well with a variety of data sets, fully displaying its great predictive ability. The predictions of four DL algorithms and two traditional models are contrasted with the test dataset of actual deaths in Figure 15. This graph’s *y*-axis shows how many people die in California each day. The final two months of 2021, March and April, are represented by the *x*-axis. Additionally, Figure 16 compares the four DL algorithms and two traditional models by projecting date ranges using the test dataset from New York. The daily death toll in New York is shown on the *y*-axis. Figure 17 compares four DL algorithms and two traditional models by forecasting using the date ranges from the VA test dataset. The total number of VA fatalities is shown on the *y*-axis. Our model is unquestionably the best option here, as it attempted to capture the peaks, which is critical in COVID-19 predictions.

### 5.4. Forecasting the Number of Administrated Vaccine Doses

**Figure 18:**
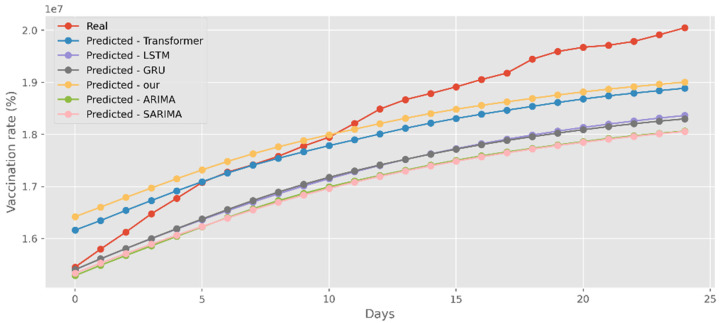
Comparison of COVID-19 prediction models of vaccinated in CA.

**Figure 19:**
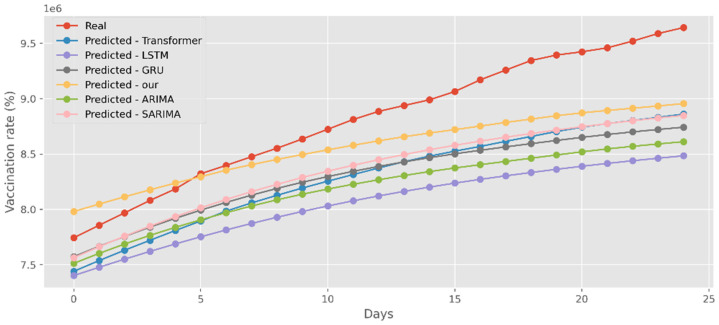
Comparison of COVID-19 prediction models of vaccinated in NY.

**Figure 20:**
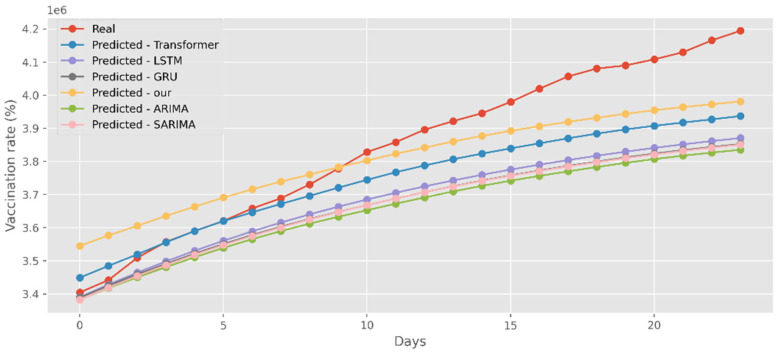
Comparison of COVID-19 prediction models of vaccinated in VA.

Our model not only has better predictive results in terms of the number of deaths and confirmed diagnoses, but it also has a better fit in terms of vaccination rates. Figure 18 displays calculations based on a real-world vaccination dataset using four distinct DL methods and two traditional models. The cumulative number of immunizations in CA is shown on the graph’s *y*-axis. The most recent 25 days are shown on the *x*-axis. Additionally, Figure 19 compares four DL algorithms and two traditional models by forecasting date ranges using the test dataset for New York. The cumulative immunizations for New York are shown on the *y*-axis. The VA test dataset’s date ranges are utilized in Figure 20 to compare four DL algorithms and two traditional models. The VA vaccinations are shown on the *y*-axis. Our model and Transformers are the best options since they are the most similar to the real-world test dataset.

It can be seen from the above analysis that the proposed model has a better predictive performance than the DL models and the traditional models. The model in this paper can fit more accurate functional equations by complex nonlinear functions and train the model with low time complexity, which can reduce the consumption of computer resources in ensuring accuracy.

## 6. Discussions

Through the continuous development and refinement of forecasting theory and techniques, many statistical theories, forecasting methods, and forecasting models have been applied in the forecasting of infectious diseases. Infection forecasting plays an important role in the prevention and control of infectious diseases and is the basis for the effective prevention and control of the development of infectious diseases. In this paper, we propose a hybrid model based on an improved Transformer and GCN for COVID-19 forecasting. The salient feature of the model in this paper is that rich temporal sequence information is extracted by the multi-head attention mechanism, and then the correlation of temporal sequence information is further aggregated by GCN. In addition, in order to solve the problem of the high time complexity of the existing Transformer, we use the cosine function to replace the softmax calculation, so that the calculation of K, Q, and V can be split, and the time complexity is reduced from the original $O(N^{2})$ to O(N). The experimental results show that our model surpasses the existing base model of DL in terms of prediction accuracy, fitting effect, and convergence.

The COVID-19 genome is still changing as the epidemic spreads because it is an emerging infectious disease. According to recent research, certain mutated viruses are more contagious as society develops economically, the environment changes, and prevention and control strategies are implemented, as well as other factors. Also varied are the COVID-19 transmission factors. Thus, it is of the utmost importance to include more influencing factors and create multi-factor prediction models based on more complete and accurate time series data, which can also support the prevention and control of COVID-19 in other nations/regions and offer concepts for emerging infectious disease research in the field of public health.

## Figures and Tables

**Table 1:** The structure of the cases and deaths dataset.

Date	State	Cases	Deaths
2021/4/22	Ohio	1,060,119	19,033
2021/4/22	Oklahoma	446,246	6716
2021/4/22	Oregon	178,110	2484
2021/4/22	Pennsylvania	1,128,144	25,934
2021/4/22	Puerto Rico	158,827	2238
2021/4/22	Rhode Island	146,028	2660

## Data Availability

https://www.kaggle.com/datasets/sudalairajkumar/novel-corona-virus-2019-dataset (accessed on 20 August 2022).
